# Integrating genomic medicine into primary care –examining perceptions of community advisory board members

**DOI:** 10.1007/s12687-026-00885-9

**Published:** 2026-06-17

**Authors:** Paramita Das, Whitley V. Kelley, Catanya G. Stager, Tiffany Osborne, Irene Moss, Kelly East, Samantha Whitfield, Bruce Korf, Nita Limdi, Lori Brand Bateman

**Affiliations:** 1 Heersink School of Medicine, University of Alabama at Birmingham, 701 19th Street South, Birmingham, AL 35294 US; 2 HudsonAlpha Institute for Biotechnology, Birmingham, AL US

**Keywords:** Community advisory board, Genomic medicine, Qualitative research, Genetic testing, Primary care

## Abstract

The Alabama Genomic Health Initiative (AGHI), funded by the state of Alabama, aims to provide genomic testing, interpretation, and counseling free of charge to Alabama residents. A 14-member Community Advisory Board (CAB) was convened in 2021 to provide community insight on integrating genomic medicine into clinical care and assessing future perspectives. The CAB was comprised of members from diverse ethnic backgrounds and represented a wide range of age groups, with balanced gender representation from two Alabama counties. The CAB met quarterly, and the team explored the perceptions of members related to genomic medicine through qualitative inquiry with exploratory quantitative findings. Self-administered pre- and post-surveys, completed prior to the first CAB meeting (pre) and after meeting five (post), were utilized to evaluate positive and negative views towards the future of genomic medicine. Five meetings were conducted in a focus group format, and transcripts were coded and analyzed to identify emerging themes. Ten of fourteen (71%) CAB members completed both surveys. Results indicate there was a slight shift in responses related to the future of genomic medicine from pre to post-test, but no significant changes were noted. Eleven of fourteen (80%) CAB members attended four out of five meetings. The prominent themes included barriers to genomic testing, strategies for recruitment, and recommendations for sharing test results. Members believed that participation in the CAB facilitated acquiring new knowledge and insight on genomic medicine.

**Supplementary Information:**

The online version contains supplementary material available at https://doi.org/10.1007/s12687-026-00885-9.

## Introduction

Our genomes provide valuable insights into our risk for certain diseases and conditions (Guttmacher and Collins 2002). For example, risk factors for heart disease, diabetes, cancer and Alzheimer’s disease can be assessed through genetic testing, which provides an opportunity for healthcare providers to identify individuals with higher risk before the onset of symptoms and take steps to tailor their treatment plan for better health outcomes (Khoury et al. 2012). Understanding risk factors about genomic medicine is clear for monogenic conditions like sickle cell anemia, cystic fibrosis, Huntington’s Disease, and Tay-Sachs disease. Further, pharmacogenomics and precision medicine have predicted drug efficacy rates as well as adverse drug reactions for different populations (Pirmohamed 2023). While still present, the benefit of participating in genomic medicine is less clear with polygenic conditions (e.g., Type 2 Diabetes, heart disease, hypertension, obesity, and certain cancers) (Fahed et al. 2020). Polygenic risk scores can help identify individuals who would benefit from personalized strategies in treatment decision making (Wray et al. 2021) even though the research on the benefits of polygenic risk scores for populations not of Northern European ancestry is still nascent (Duncan et., 2019). Even while genetic testing is being incorporated as part of typical healthcare practice (Sherburn et al. 2023), the public knowledge of genomics literacy remains low (Nikitara et al. 2025; Calabrò et al. 2024).Further, genomic medicine has revolutionized healthcare to be more precise and personalized with enhanced treatment outcomes, reducing adverse reactions of medications in patients. Importantly, genomic medicine helps to reduce healthcare costs by avoiding unnecessary treatments and medications.

Early detection of disease and targeted interventions can lead to reduced morbidity and mortality, and in implementing genomic health for all, barriers such as access to genetic testing, privacy concerns and ethical considerations must be addressed to ensure equitable benefits across populations (Fogleman et al. 2019). However, individuals from underserved communities are less likely to receive genetic testing and counseling, which could delay diagnosis and treatment of genetic conditions (Tawfiket al. 2023). Because of a lack of access to testing, coupled with the fact that certain populations may be more genetically predisposed to specific conditions and diseases (Isleret al. 2013), genetic factors, along with factors such as socioeconomic status and healthcare access, may play a significant role in contributing to health disparities in vulnerable communities (Kashyap et al. 2015). This may be particularly applicable to racial and ethnic minority groups who may experience disparities in treatment and outcomes due to reasons ranging from lack of services to their negative perception about health services (González 2012; Halbert 2022). In this context, genomic health initiatives have the potential to improve health outcomes, reduce health disparities, and reduce healthcare costs.

As a joint effort between the University of Alabama at Birmingham (UAB), HudsonAlpha Institute for Biotechnology, and Tuskegee University, the **Alabama Genomic Health Initiative (AGHI**) was launched in 2017 to provide genomic testing, interpretation, and counseling free of charge to Alabama residents (Limdi et al. 2023). AGHI is one of the nation’s first statewide efforts to harness the power of genomic analysis and aims to ensure access to healthcare, reduce health disparities and improve health outcomes across the population (Alabama Genomic Health Initiative) (Heffernan et al. 2026). From the beginning, community engaged research strategies have been utilized to ensure community perspectives are integrated into the project (Hahn et al. 2010; May et al. 2020; Zierhut et al. 2024). This includes recruiting and engaging a diverse group of participants (Institute 2017). The community advisory board (CAB) was launched in the summer of 2017 to oversee patient participant recruitment strategies by offering guidance to increase visibility, understanding and the provision of statewide support for this initiative (May et al. 2020). The CAB was effective in ensuring diverse recruitment, evaluating recruitment materials, and helping the program team understand the need for a simplified return-of-result process. CAB members also recommended strategies to improve enrollment of African American participants by engaging faith-based organizations and conducting outreach at community health fairs. As a result, in 2020 AGHI was able to achieve an overall representation exceeding 20% for the African American population, which approaches the proportion of African Americans in the population of Alabama (26.6%) (May et al. 2020).

In 2021 AGHI was relaunched and adapted from a participant facing model of service delivery to a model of integrating services into primary care settings and equipping health care providers to increase participation and engagement (Korf et al. 2022). Two UAB primary care clinics in Jefferson County and one in Dallas County were selected to integrate AGHI into their services. Currently, AGHI’s patient care model comprises genomic testing, interpretation, genetic counseling, and pharmacogenetics analysis of current and future medications for all registered patients/participants. Investigators wanted to provide increased access and representation to the study for underrepresented groups such as populations historically lacking in genomic data. To that end, the new CAB included representative key stakeholders and leaders from community-based organizations in both counties. One key goal of the new CAB, was to serve in an advisory role and ensure community perspectives are taken into account in all aspects of the project including participant recruitment, communication, the return of results process and impact on participants receiving results.

In this study, CAB members were engaged to provide their insight on project goals, implementation, project materials, and dissemination strategies in the light of previous CAB’s broad recommendations and the current post-COVID context. In effect, the CAB served as a small-scale focus group of stakeholders and representatives for consultation, to provide community insight into what the community needed for genomics implementation. Self-administered pre-/post-surveys and focus groups were conducted with the CAB to understand their perceptions of (1) integrating genomic medicine into the primary care setting, and (2) the future of genomic medicine from the community perspective.

## Methods

AGHI was launched into three UAB primary clinics located in the Jefferson (2 clinics) and Dallas (1 clinic) counties, and community champions representing each county were invited to join the CAB. A new CAB was formed in 2021 that included community champions from diverse Alabama populations living in Jefferson and Dallas Counties. These clinics were chosen because UAB has a strong presence in both the counties in rendering primary health care services to the residents. Dallas County is in the rural Black Belt where 29% of residents live below the poverty line and face limited healthcare access due to provider shortages and transportation barriers. With a population that is 71% African American, residents rely heavily on local public health clinics and federally qualified health centers for essential care. Jefferson County is largely urban and suburban, centered around Birmingham, with a diverse population that continues to experience persistent socioeconomic and health disparities. Although residents have access to advanced medical facilities such as UAB Medicine and Children’s of Alabama, ongoing inequities still limit healthcare access and insurance coverage for underserved communities.

Eligible participants for the CAB were selected based on their county of residence (Jefferson or Dallas) and involvement in community work. We sought a CAB that would be gender-balanced and representative of the populations in each county. Each invited community member was an advocate and/or representative of a local faith-based, social and civic community organization, neighborhood association, healthcare organization, government agency, or business. In late 2021, the 14-member CAB was established, which consisted of 7 men and 7 women, aged 35–66 years, and represented the race/ethnicity of African American (5), White (4), Latino/Latina (2), and Unknown (2). Two of the CAB members expressed experience with some level of genomic testing: one individual, with a medical background, completed a personal genetic test with a private company and another reported familial genetic testing due to a rare genetic blood cancer. The remaining members shared no experience with medical genetics.

The study was conducted between December 2021- June 2023 and was approved under IRB.

IRB-170,303,004. CAB members were invited to participate in the study and verbal consent was obtained. A primarily qualitative approach with exploratory quantitative methods was used to explore perceptions related to genomic medicine during each quarterly, virtual CAB meeting, which were conducted using a focus group format. Baseline surveys were conducted prior to the first CAB meeting and follow-up surveys conducted after five consecutive CAB meetings. The survey instrument was based on the validated Genomic Orientation (GO) scale (Horrow et al. 2019), which examined positive and negative views (optimism and pessimism) related to the future of genomic medicine (for a list of concepts included in each subscale, please see Fig. 1). The GO scale survey was administered at two time points, as a pre-test and a post-test. Fourteen CAB members completed the pretest, and 10 CAB members completed the post-test administration of the GO scale survey This survey was sent electronically to each of the participants prior to the first CAB meeting. Measuring attitudes towards genomics can help provide insight into the optimism (Kang et al. 2023) toward implementation of genomics into the healthcare system (Horrow et al. 2019) regardless of public levels of genomics literacy (Nikitara et al. al., 2025; Calabrò et al., 2024), Thus, genomic orientation/attitudes scores may provide insight into changes in the CAB’s perceptions toward implementing genomics in their communities. Although our sample size was too small to test for significance, we incorporated the survey to observe trends. Five virtual focus group discussions (FGDs) were conducted by the AGHI team during the CAB meetings to understand community perspectives of integrating AGHI into the primary care setting. Before each focus group discussion began, participants were provided an overview of the topic, which often included case studies. Participants completed the follow-up survey after the last FGD.

FGDs lasted approximately 90 min and were audio and video recorded. The AGHI research team developed a semi-structured focus group guide for each FGD, and a facilitator (part of the AGHI community engagement team) and a content expert (AGHI genetic counselor) jointly conducted each session. Each of the five FGDs had an assigned unique topic for discussion (See Table 1 for the list of meeting topics across all FGDs), with content including introductory and opening questions; transition questions; approximately 10-minute presentation on the topic; questions about potential facilitators and barriers related to the topic; and approximately10 minutes of summary. Participants were allowed to veer off the guide and discuss related topics. A $50 incentive was provided for participation in each FGD. While all CAB members attended a FGD throughout the study period, there was not a focus group discussion where all 14 CAB members were in attendance.

**Table 1:** Meeting topics

Meeting Date	Meeting Topic	CAB members in attendance
December 7, 2021	Genetic Testing	13
February 24, 2022	Recruitment	7
June 16, 2022	Test Results	12
October 10, 2022	Family and Test Results	11
June 15, 2023	CAB Experiences	10

### Analysis

#### Quantitative analysis

For the GO scale survey, responses to the 21 questions (5-point Likert scale) were analyzed to assess the subscale factors of pessimism and optimism towards the future of genomic medicine. Each item asked for a response based on a 5-point Likert scale: “very unlikely,” “unlikely,” “neither likely nor unlikely,” “likely,” and “very likely.” Response options were coded from − 2 to 2. Response distribution indicated changes from pre- to post-test, but no significance testing was performed due to the limited small sample size.

#### Qualitative analysis

FGD transcripts were audio-recorded, transcribed, and analyzed by the research team using deductive thematic analysis (TA) and NVivo 12 (Lumivero 2025) to organize the data. (While the interview guides were adapted based on earlier FGDs, the FGD transcripts were coded only after the completion of all meetings. The CAB participant response analysis was conducted on aggregated responses to all of the five topics) TA can be done deductively or inductively and enables researchers to identify emerging codes and primary themes. TA consists of familiarization, coding, searching for theoretically-drive themes, reviewing themes, and clarifying and/or articulating thematic definitions (Clarke et al. 2015; Braun and Clarke 2016, 2021; Clarke and Braun 2021). Our team began with the interview guide to develop an initial codebook. From there, two researchers read and coded the transcripts independently, adding additional sub-codes that emerged in the data, and then agreed upon the final code list. To ensure reliability, validity, and rigor, each transcript was then coded by a primary and a secondary coder. Next, analysts identified emerging themes that were present across the transcripts. Themes identified by several participants, across more than three FGD sessions were included as major themes. When needed, coding disagreements were resolved by an independent third coder.

## Results

### Quantitative analysis

Fourteen CAB members completed the pretest, and ten CAB members also completed the post-test surveys. All analyses on the GO scale were conducted with only those participants who completed the pre- and the post-test administrations of the GO scale survey (*n* = 10). Our quantitative findings are exploratory due to our small sample size, and we report our findings with caution. Post-test responses (*n* = 10) to individual questions indicated that following the five FGDs, CAB members agreed (likely or very likely) with a positive view of genomic medicine. Noteworthily, all CAB members (10 of 10) agreed that in the next five years, genomic medicine would improve their health, help diagnose rare diseases earlier, help doctors choose the best drugs for patients, help researchers answer difficult disease questions, and help individuals live longer. 90% of CAB members agreed that genomic medicine would help researchers find new treatments for common diseases while 80% of CAB members agreed that genomic medicine would improve the quality of health care, benefit nearly all aspects of medicine, and give individuals more control over their health.

In our sample, the pessimism subscale score had a pretest mean of 0.34, (SD = 0.49, range − 0.40 to 1.40) and a posttest mean of 0.20 (SD = 0.52, range − 0.80 to 1.20). The decrease (-0.14) indicates a decrease in pessimism. The optimism subscale score had a pretest mean of 1.05, (SD = 0.54, range 0.31 to 2.00) and a posttest mean of 0.88 (SD = 0.52, range − 0.06 to 1.75). The decrease (0.17) indicates a decrease in optimism. Overall, toward the future of genomics, respondents indicated a decrease in pessimism (less negative attitudes toward negative outcomes after the CAB meetings) and a decrease in optimism (less positive attitudes toward positive outcomes) (See Discussion for details). The range of scores across all questions can be seen in Fig. 1.

Fig. 1Distribution of responses (Panels **A **[Pretest] and **B** [Post-test])

**Figure:**
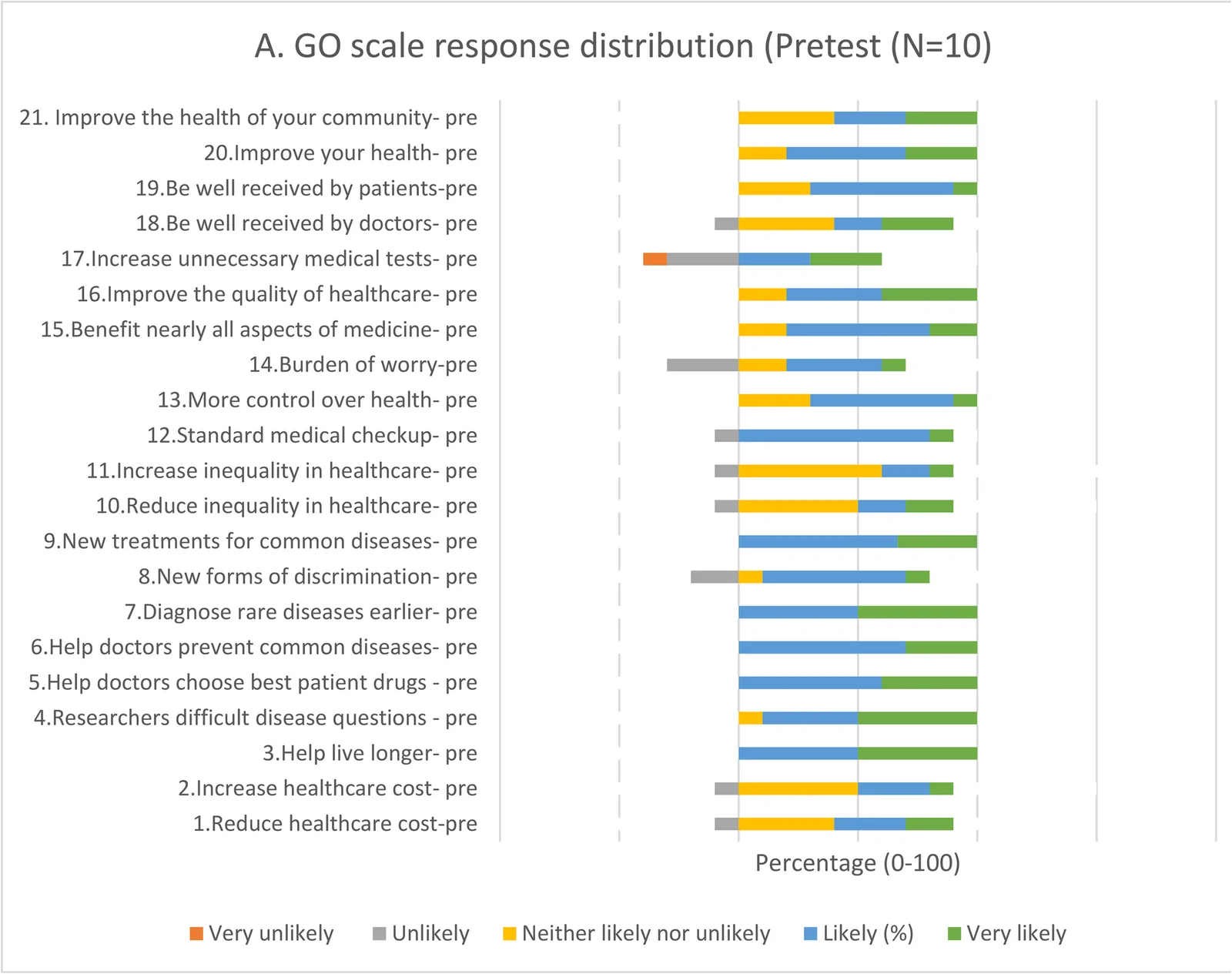

**Figure:**
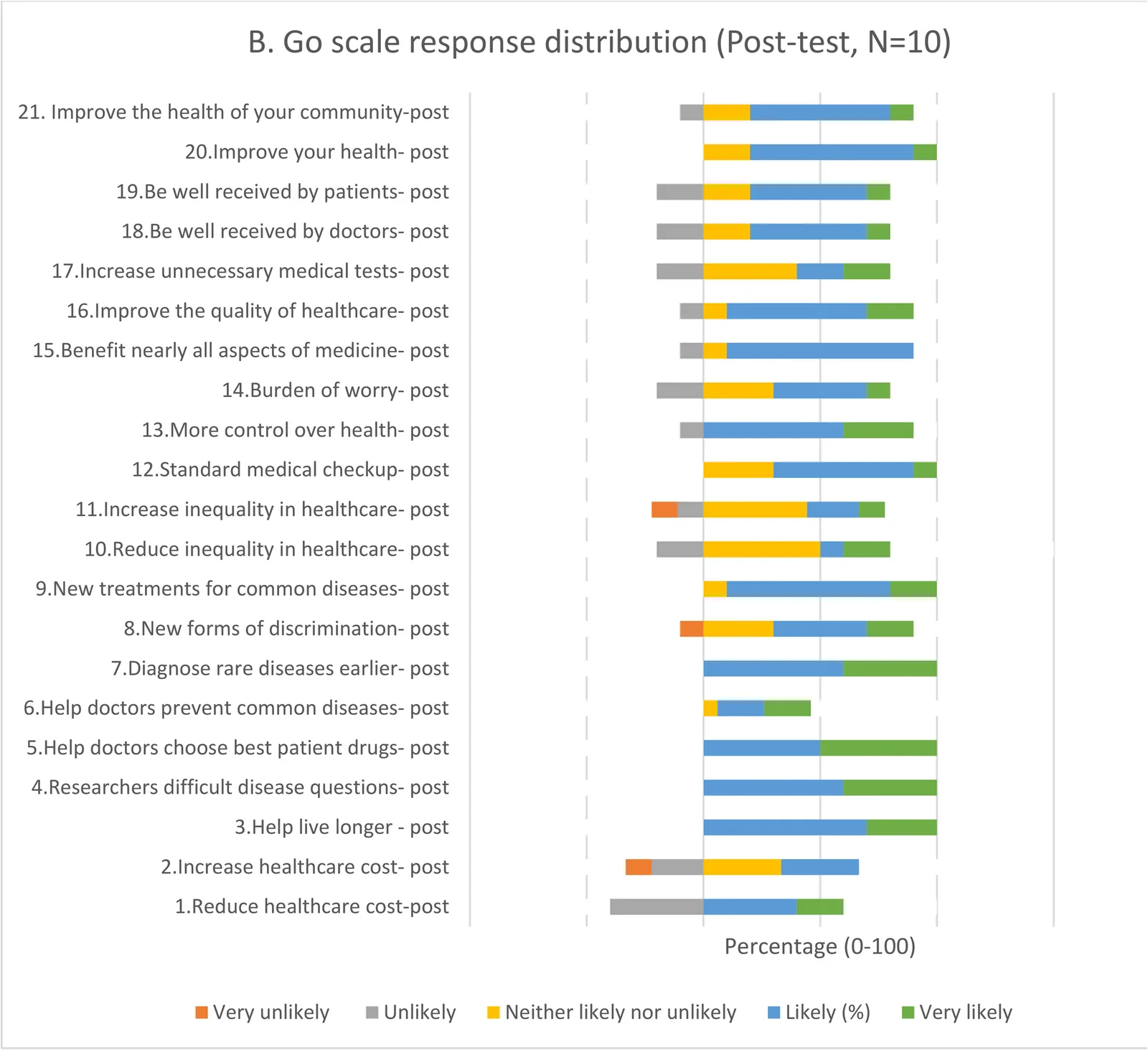


### Qualitative analysis

80% of CAB members attended 4 of 5 meetings/FGDs and provided their feedback on each of the topics discussed. Primary themes include barriers to genomic testing, insurance implications, clinic-based recruitment, and the process of receiving genetic test results.

#### Barriers to testing

. Though most CAB members were aware of genomic medicine and its benefit to improve health outcomes, they were concerned about genomic testing. Almost all members provided feedback regarding barriers they perceived as major hurdles in accessing genomic testing by certain communities. Three sub-themes emerged in the main theme of barriers to testing including trust, fear of the unknown and accessibility to testing.

When asked about participating in genomic testing, one CAB member expressed hesitation due to concerns about trust, “*I wouldn’t take the test*,* I know*,* just because in my community what our relationships have been and how we’ve been treated when we do go to the doctor……….*,* so*,* for me*,* just because of that relationship*,* my personal relationship with healthcare*.” This quote represents mistrust in the healthcare system and in the lack of healthcare provider - patient communication, which poses as a barrier in accessing genomic testing. Some CAB members required more information before they could decide whether to have tests done or not. To overcome this long-standing mistrust, a CAB member recommended focusing on relationships: “*There is a very important need for continuous and long-term trust building.*”

Another barrier identified by CAB members to genetic testing was the fear of the unknown, as seen in this quote: “*I wouldn’t take the test because of the simple fact I would worry myself to death trying to figure out what’s gonna happen……*”. Participants raised concerns that “they wouldn’t take the test” and didn’t need to know about getting health information through genetic testing as they feel scared, uncomfortable and emotional about it. One member put it this way, “*I think that’s gonna be huge. I think that’s even bigger than we realize. The emotional part I think is gonna outweigh*.”

For other CAB members, access to genomic testing remains as a barrier due to lack of medical services in certain areas, as one member stated, “*we’re learning that there is a medical service desert in the underserved communities as well.*” While this was an important issue that needs more attention, the CAB members also offered solutions to address this issue: “*I’m on the board of a federally qualified health center*,* but have you considered partnering with some of the federally qualified health centers because they are probably the best able to reach into some of these underserved communities you’re talking*.”

#### Insurance implications

Among the questions participants asked during FGDs, insurance remained a top priority, especially regarding eligibility. “*Now will the life insurance consider that as being somewhat of a screening and not really affect the eligibility on life insurance*,* or do we not really know*?” Participants raised concerns on privacy and genetic discrimination based on the test results with queries like “*Are insurance companies allowed to do genetic testing of their own against people*?”

#### Recruitment of participants

Though one focus group session was dedicated to discussing recruitment strategies, we found participants feedback on this topic in 3 of the FGDs. When asked about recruitment, most respondents recommended adopting varied strategies to facilitate and encourage various groups and communities to participate in genomic medicine. Participants suggested involving primary care doctors to educate and inform patients: “*I think it would because most people are comfortable talking to a doctor…A recommendation from their doctor*,* if they were hesitant*,* I think that would be helpful*.”

A few respondents mentioned using printed educational materials written in simple language to generate awareness while others talked about developing an all-out media strategy involving video and television commercials. “*I think considering the effort of partnering with the media*,* the local Hispanic media*,* like Latino News*,* La Jefa*,* inviting them*,* one of the person that is an expert*,* inviting the community and explaining them what is this process about….”*. There were comments on television commercials as well. “*Those are 30 seconds and also 60 seconds PSAs that we need to be running for—if we can identify the target audience*,* …”*.

Nearly all participants noted the importance of long-term efforts to motivate certain groups and communities to participate in genomic medicine in the primary care setting, with a focus on community. As one participant described it, “*Also*,* to build community. As we’re getting more people involved*,* as we’re gettin’ people connected with the initiative and they’re going through the process there should be some type of way that we’re building community*,* consistently reaching out*.”

#### Receiving test results

When asked about best ways to receive test results, participants’ feedback ranged from psychological impact related to receiving stressful health news to proactive role of the primary care provider. Respondents presented their viewpoints on this topic throughout the different scheduled FGDs. One participant noted, “*Because when we become emotional*,* we usually become vulnerable when we open up*,* so if somebody is gonna share news with me that are distressing*,* do we really need to have somebody who brokers that relationship and supports the emotional part before we get into the nitty-gritty of the science?*”

There was an overwhelming consensus among the respondents that the genetic test results ideally should be received in a joint session with the PCP and genetic counselor. As an example, one participant suggested, *“…a joint session with a primary care provider that the patient knows and trusts and maybe one of your genetic counselors zooming into that meeting might be a way to go ahead and take care of both the medical and the psychological parts of it*.”

While participants mentioned barriers, nearly all respondents shared positive feedback being on the advisory board. They revealed that participating in FGDs helped increase their knowledge of genomic medicine and encouraged them to begin conversations in their families and communities.

## Discussion

The current study examined CAB members’ perceptions related to barriers and facilitators to implementing genomic medicine in underserved communities. Our study found that genomics-centered focus groups for the CAB did strengthen community attitudes toward genomics and that CAB members had a predominantly positive view toward the future of genomics. FGDs further validated the impact of the genomics focus groups on CAB members’ attitudes toward implementing genomics in community settings.

While other studies have conducted community surveys and interviews to optimize genomic medicine implementation (Matthews et al. 2018; Connors et al. 2019; Vidgen et al. 2022), our genomics implementation study has been uniquely grounded in the principles of Community-Based Participatory research from the onset, with our study collaborating to engage and empower community stakeholders to help identify barriers and facilitators and best practices in implementing genomic screening in primary care. Through a 16-month long series of 5 FGD, our CAB members provided their local insights on project goals, implementation, project materials, and dissemination strategies for their communities, and their unique insight will be instrumental to optimizing the implementation of genomic medicine in communities. Stakeholder responses are also helping to inform the continued process of implementing genomic medicine with care providers in underserved communities. For example, one participant response about partnering with a federally qualified health center to reach underserved communities resulted in a partnership with a federally qualified health center to explore provider views of barriers and facilitators to implementing genomic medicine in their clinic.

Previous genomic studies have shown financial considerations, such as insurance and cost, as barriers to genetics in community settings (Fogleman et al. 2019) as well as ethical considerations, like mistrust and over-reaching of science (Isler et al. 2013; Tawfik et al. 2023). Our quantitative findings were exploratory because of our small sample size, but our qualitative component of the study was able to dive deeper into the barriers toward genomic medicine. We identified themes of healthcare mistrust, fear of the unknown, and limited healthcare access as barriers, in line with previous studies (Halbert 2022). Our study also explored CAB concerns about the potential stress with receiving genetic testing results and ways to mitigate those stressors.

Psychological distress appears as personal anxiety toward the uncertainty of genomic information (Taber et al. 2015). While cancer patients offered genetic testing report anxiety toward potentially gaining personal genetic knowledge (Austin et al. 2025; Smith-Uffen et al. 2021; Leventhal et al. 2013), some patients would rather remain ignorant than be tested (Birmingham et al. 2013). Our CAB (non-patient) sample expressed that fear (anxiety) of the unknown was a barrier to genomics testing in primary care settings for their communities. It may be that this psychological barrier could be partially mitigated by addressing factors such as testing logistics (cost, insurance), stigma, and genetic testing knowledge (Austin et al. 2025; Hann et al. 2017).Studies have found that an understanding of the utility of genomic medicine, genetic literacy, and community buy-in are necessary facilitators for integrating genomic medicine into practice (Shen et al. 2022; Tawfik et al. 2023). In our study, nearly all of our CAB members indicated at post-test that genomic medicine was of benefit to themselves, their community, and overall healthcare, similar to other studies (Fogleman et al. 2019). Our CAB also reported that recruiting should be integrated across demographic groups, in agreement with studies that focus on psychosocial and cultural factors as resources for health equity in genomics testing (Halbert 2022). The importance of long-term efforts to motivate certain groups and communities to participate in genomic medicine in the primary care setting was mentioned by nearly all participants. One of the key goals of AGHI since its founding in 2017 has been to build strong relationships with key stakeholders in representative communities in Alabama to assess recruitment, education, and communication strategies. Strategies have included the establishment of a Engagement Working Group to serve as a liaison, to communicate needs of communities to the broader AGHI leadership team, town hall meetings, outreach events, and facilitated deliberative process (FDP) groups.( May et al. 2020).

Participants described the psychological impact related to receiving stressful genetic test results and there was an overwhelming feeling that the results would ideally be returned in a joint appointment with both a PCP and genetic counselor. Evidence indicates that embedding genetic counselors may be an effective way to address health care access disparities, enhance the delivery of genomic medicine, and improve patient outcomes (Pan et al. 2025; Mayo Clinic 2026), However, studies indicate that current barriers to integrating genetic counselors into primary care settings may include difficulties related to integration into the care team, perceived lack of buy-in at the multiple levels including among decision makers, and a lack of resources (Weiss et al. 2025). More research is needed to study the integration of genetic counselors into the primary care setting (Mayo Clinic Proceed 2026; Parente 2022).

Curiously, when we examined changes in pre- and post-test factor scores of the GO scale survey, overall optimism showed a small decrease, Because of our small sample size, we cannot read much into these findings. Studies examining scientific optimism indicate that as the perceptions of risk and public distrust increase, optimism decreases: positive assumptions on possible technology benefits may initially over-inflate levels of optimism in new technology (Clark and Hampton, 2016), based on changing perceptions of risk (Hochschild et al. 2012) and institutional trust (Kang,. Vedlitz, Goldsmith, and Seavey, 2023),

Limitations in this study include our small sample size (quantitative findings), the fact that all FGDs were conducted with the same group of participants, and the lack of participants under the age of 35. Other studies should also consider how to obtain matched pre-test and post-test from community members, as community member attendance was uneven across the focus groups. However, despite these limitations, we feel this research adds to the literature on community perspectives related to barriers and facilitators to the uptake of genomic medicine. Future researchers should continue to study attitudes and behaviors toward genomic testing across populations to understand how individuals might translate genetic literacy into participating in genomic medicine as recommended by their provider to enhance their own health. Future studies should also consider how CABs can be empowered to work with healthcare providers to implement genomic medicine in their communities.

## Conclusion

This study highlights the perceptions of CAB members in identifying barriers and facilitators related to patient participation in genomic medicine, with the ultimate goals of improving health outcomes and reducing health disparities. Quantitative analysis suggests that CAB members’ perceptions related to genomic medicine may have changed by attending FGDs. Qualitative analysis identified trust, accessibility to testing, and fear of the unknown as barriers to genetic testing in diverse communities. To improve community participation in genomic medicine, this study suggests that involving primary care providers in test result sharing and commencing awareness campaigns about the benefits of genomic medicine could be beneficial. Future research should continue to explore the perspectives of diverse community members in the planning, implementation, and evaluation of genomic medicine initiatives.

## Electronic Supplementary Material

Below is the link to the electronic supplementary material.


Supplementary Material 1


## Data Availability

The data that support the findings of this study are available from the corresponding author upon reasonable request.
