# Supplementary material for: Integrating genomic medicine into primary care –examining perceptions of community advisory board members
Source: J Community Genet. 2026 Jun 17;17(4):76. doi: 10.1007/s12687-026-00885-9 (PMC13272711; doi:10.1007/s12687-026-00885-9)
Supplement: Supplementary file 1 — Supplementary Material 1 [file 12687_2026_885_MOESM1_ESM.docx]

Supplementary File to

“Integrating Genomic Medicine into Primary Care – Examining Perceptions of Community Advisory Board Members”

Alabama Genomics Health Initiative (AGHI)

Community Advisory Board (CAB)

**Focus Group Discussion Guides**

**(2021-2023)**

**Table of Contents**

Session 1: Genetic Testing (December 2021) ………………………………..2

Session 2: Recruitment (February 2022) ……………………………………….3

Session 3: Test Results (June 2022) ……………………………………………..4

Session 4: Family and Test Results (October 2022) ………………………..6

Session 5: CAB Experiences……………………………………………………….8

**Session 1: Genetic Testing (December 2021)**

**Agenda (Guide)**

- **Read IRB‑approved information sheet**
- **Introductions**:
  - Name, professional/community role(s)/interests
  - Opening question (icebreaker)
- **Introduction to AGHI**
  - Study description and return‑of‑results workflow
  - Current enrollment data
- **Basic Genomics Concepts and Terminology**
  - Gene, variant, and related terminology
  - Limitations of current testing approaches
  - Definition of “medically actionable” findings and clinical implications
    - Include examples of possible results
- **Introductory Question**
  - What are board members’ initial impressions of AGHI (positive or negative)?
- **Transition Question**
  - What comes to mind when someone mentions “genetic testing”?
- **Key Questions**
  - What information would be most useful when initially receiving results?
    - Preferred method of delivery (in‑person, phone, letter, etc.)
  - What support or resources would be desirable after receiving results?
- What information about your genomic test result would you expect to receive from your primary care physician in this setting?
  - What role would you expect or want your PCP to have in disclosing the result and providing downstream support and resources?

**Session 2: Recruitment (February 2022)**

**Agenda (Guide)**

- **Opening Question**
- **Study Updates**
  - Current enrollment and RoR data
  - Upcoming symposium
- **Presentation of key experience or issue current to the study: Recruitment**
  - Description of recruitment process
  - Review of consent form
- **Introductory Question**
  - What are board members’ initial thoughts about the AGHI recruitment process (positive or negative)?
- **Transition Question**
  - Do you think that having recruitment for a research study takes place within the context of clinical care (i.e., at a patient’s regular doctor’s office) improves or harms patient trust? Why or why not?
- **Key Questions**
  - How might the recruitment team better communicate the difference between this research study and the clinical care provided by their doctor/the doctor’s office?
  - What are some possible ways to improve the recruitment process, especially in terms of its appeal to male patients and/or other underrepresented groups?
  - What additional communication, if any, would you expect from your doctor either before or after study recruitment if you were recruited into a study like AGHI at your own physician office?
- **Additional Questions**

**Session 3: Test Results (June 2022)**

**Agenda (Guide)**

- **Opening Question**
  - Icebreaker
- **Study Updates**
  - Current enrollment and RoR data
- **Case Presentation: See below**
- **Introductory Questions**
  - What are some good and bad experiences you have had when receiving distressing or upsetting news (medical news or otherwise)?
  - What are some ways the person(s) giving you this news or information could have better supported you/what are the ways they appropriately supported you?
- **Transition Question**
  - Would you anticipate receiving medical information *about genetic risk* would be distressing?
- **Key Questions**
  - How would you feel if you were the patient in this case?
    - **Probe:** Would you prefer phone disclosure followed by a specialist referral? Would you prefer to receive information by phone from a genetic counselor to be referred to a specialist appointment with very little explanation given?
  - What, if any, gatekeeping is appropriate for a physician to display in this setting?
  - What support, resources, or information might make the physician and participant feel more comfortable receiving these results?
  - How, if at all, should the study consent process reflect the possibility of this potential participant distress?
- **Case Studies: Examples of information that can be learned from AGHI disease risk results**
- A genetic variant was found that increases your risk to develop breast, ovarian, pancreatic, prostate, and/or melanoma cancers. Screening for breast and prostate cancers is excellent, but surgery will likely be required to manage ovarian cancer risk. Screening for pancreatic cancer is not very effective. You likely inherited this variant from a parent, and your children and siblings each have a 50% chance of also sharing this variant.
- A genetic variant was found that increases your risk of having a dangerous, possibly fatal reaction to certain anesthesia medications. If this information is communicated in your medical record and through the use of a medical alert bracelet, your doctors can avoid these triggering medications during surgical procedures, as there are other safe alternatives. You likely inherited this variant from a parent, and your children and siblings each have a 50% chance of also sharing this variant.
- A genetic variant was found that increases your lifetime risk of having colon cancer to nearly 100%, due to an overgrowth of colon polyps. The best way to address this risk is to have your colon removed in early adulthood. It is possible that this variant is brand new in you (not inherited from a parent, meaning siblings would likely not be at risk), but each of your children would have a 50% chance of inheriting this variant. If they share this variant, they would need to begin having colonoscopies in childhood to check for an overgrowth of polyps, and they would likely need to consider having their colon removed in adulthood.
- A genetic variant was found that increases your risk of having or developing a heart muscle problem that may cause early heart failure and/or sudden cardiac arrest. Tests can determine whether you currently have the heart muscle problem, but not having evidence of it now does not mean you will not develop it later; long‑term screening is typically needed. You also may never develop symptoms. Medications, lifestyle changes, and procedures (such as placement of a pacemaker) can help manage the risk of serious complications if a heart muscle problem is found. You likely inherited this variant from a parent, and your children and siblings each have a 50% chance of also sharing this variant.

**Session 4: Family and Test Results (October 2022)**

**Agenda (Guide)**

- **Opening Question**
  - Icebreaker
- **Study Updates**
  - Current enrollment and RoR data
    - Provide numbers only; avoid demographic data unless major shifts occur
- **Participant‑Facing Family History Process**
  - Overview of questionnaire
  - Examples of report text
- **Introductory Questions**
  - What discussions have you or others had with healthcare providers about family history?
    - How is the information elicited?
  - What actions have providers taken based on family history?
    - Yes/no responses acceptable
- **Transition Question**
  - If actions based on family history were taken, did you feel comforted that those actions actually reduced your risk of developing a serious problem? Please explain further, if you feel comfortable doing so.
- **Key Questions**
  - If you received a result from the AGHI (or any other medical testing) like the above example(s), what would be your initial reaction to the family history risk assessment section?
  - If you received a result, can you describe how you would like your provider to respond?
  - Should they bring it up first

- What conversations would you expect to have with relatives?
  - - Which relatives would you contact first?
- **If Time Permits**
  - Review the family‑history triage process
    - Ask whether its intent is clear based on initial materials

**Session 5: CAB Experiences**

**Agenda (Guide)**

- **Opening Question**
  - Icebreaker
- **Study Updates**
  - Current enrollment and RoR data
    - Consider moving to end or emailing separately to avoid distraction
- **Introductory Questions**
  - What has your experience been like serving on the CAB?
  - Who, if anyone, knows you have been part of our community advisory board (family members, coworkers, friends, etc.)?
    - What types of conversations have you had with people in your life regarding your participation on our board?
- **Transition Question**
  - Have you experienced any personal impacts from participating on the board?
- **Key Questions**
  - What new concepts or ideas have you learned through the CAB?
  - Have your views on genomic testing changed?
    - If yes, how?
  - What additional issues or topics related to genomic testing in primary care should the study or board address?
- **If Time Permits**
  - Present survey data at the end of the meeting
